# Fibroblast as a critical stromal cell type determining prognosis in prostate cancer

**DOI:** 10.1002/pros.23867

**Published:** 2019-07-03

**Authors:** Sami Blom, Andrew Erickson, Arne Östman, Antti Rannikko, Tuomas Mirtti, Olli Kallioniemi, Teijo Pellinen

**Affiliations:** ^1^ Institute for Molecular Medicine Finland (FIMM), Helsinki Institute of Life Science (HiLIFE) University of Helsinki Helsinki Finland; ^2^ Science for Life Laboratory, Department of Oncology and Pathology Karolinska Institutet Stockholm Sweden; ^3^ Department of Urology Helsinki University and Helsinki University Hospital Helsinki Finland; ^4^ Department of Pathology University of Helsinki Helsinki Finland

**Keywords:** castration‐resistant prostate cancer, digital image analysis, multiplex tissue imaging

## Abstract

**Background:**

Tumor stroma associates with prostate cancer (PCa) progression, but its specific cellular composition and association to patient survival outcome have not been characterized.

**Methods:**

We analyzed stromal composition in human PCa using multiplex immunohistochemistry and quantitative, high‐resolution image analysis in two retrospective, formalin‐fixed paraffin embedded observational clinical cohorts (Cohort I, n = 117; Cohort II, n = 340) using PCa‐specific mortality as outcome measurement.

**Results:**

A high proportion of fibroblasts associated with aggressive disease and castration‐resistant prostate cancer (CRPC). In a multivariate analysis, increase in fibroblast proportion predicted poor cancer‐specific outcome independently in the two clinical cohorts studied.

**Conclusions:**

Fibroblasts were the most important cell type in determining prognosis in PCa and associated with CRPC. Thus, the stromal composition could be critically important in developing diagnostic and therapeutic approaches to aggressive prostate cancer.

## 1 INTRODUCTION

Prostate cancer (PCa) is the most common noncutaneous cancer in males globally.^1^ Normal prostate tissue consists of epithelium and stroma, which interact to maintain the physiological homeostasis of the organ. The stromal compartment comprises fibroblasts, smooth muscle cells, immune cells, and a collagen‐rich extracellular matrix that is located between the secretory prostatic acini. In tumorigenesis, the normal organ architecture is disrupted, which activates various feedback responses both to the epithelial and stromal compartments. In 1986, Dvorak^2^ described tumors as “wounds that do not heal” and suggested that the stromal cells actively interact with the cancer cells of epithelial origin. Indeed, reactive stromal cells often form a fibrotic reaction around tumors mimicking the wound healing process.^3^

Recently, stroma has been shown to have both tumor‐suppressing and tumor‐promoting effects in many solid tumors.^4^, ^5^, ^6^ Olumi et al^7^ described a dramatic tumor‐promoting effect of carcinoma‐associated fibroblasts in PCa, and tumor stroma has been shown to be associated with aggressive disease features^8^, ^9^ and shorter time to biochemical recurrence using needle biopsy^10^ as well as prostatectomy samples.^11^, ^12^ Moreover, stromal gene expression analysis in prostatectomy samples revealed signatures specific for metastatic potential of PCa.^13^

Vimentin (VIM, *VIM*) and alpha‐smooth muscle actin (aSMA, *ACTA2*) have been widely used for the classification of the different stromal cell types. VIM is expressed in fibroblasts and myofibroblasts, whereas aSMA is expressed in smooth muscle and in myofibroblasts.^14^ The growing body of evidence for the clinically significant stromal features warrants systematic characterization of the cellular composition of the cells in the tumor stroma. However, gene expression patterns or standard IHC do not allow cell‐level classification of the various cell types simultaneously in situ in human tumor samples.

Here, we performed a quantitative analysis of PCa stroma using multiplex immunohistochemistry (mIHC) and unbiased automatic image analysis to determine the different stromal cells including fibroblasts, myofibroblasts, and smooth muscle cells in situ in human PCa tissue samples. We show that a stromal phenotype with an increased number of fibroblasts associates with aggressive disease and castration resistance. The proportion of fibroblasts independently predicts poor cancer‐specific outcome in two independent patient cohorts.

## 2 MATERIALS AND METHODS

### 2.1 Patient material

Study cohorts were formalin‐fixed paraffin embedded (FFPE) samples from transurethral resection of prostate (TURP; Cohort I, n = 159 patients) and retrospective radical prostatectomy (Cohort II, n = 350 patients). Tissue microarrays (TMA) were constructed for Cohort I by taking 1 to 5 cores per patient and for Cohort II as described earlier.^15^ Prostate cancer‐specific mortality (PCSM) was recorded as end‐point in both cohorts. Cohort II included only patients who had non‐metastatic (M0) primary PCa at diagnosis and who received prostatectomy as the first treatment (excluding also neoadjuvant therapy). Phosphatase and tensin homolog (PTEN) and androgen receptor protein expression data for Cohort II were obtained from Lahdensuo et al^16^ and Sahu et al,^15^ respectively.

### 2.2 Immunohistochemistry

Antibodies used in the study are listed in Table S1. Antibodies for vimentin (VIM), alpha‐smooth muscle actin (aSMA), and CAV2 (Caveolin‐2, *CAV2*) were optimized for mIHC using immunohistochemistry in prostatectomy samples as described earlier.^17^ See full details in Supplementary Information. Antibodies for PanEpi (PanCK [clones AE3/1 and C11] + E‐cadherin (*CDH1*) [clone 36]) were optimized earlier for mIHC.^17^ The amount of VIM‐positive immune cells per patient was visually scored by SB in Cohort II as “low”, “medium”, and “high” if <1%, 1‐5%, or >5% of the stromal area was occupied by VIM‐positive immune cells, respectively, which morphologically resembled either lymphocytes or macrophages. The scoring scheme follows previously published data reporting varying immune cell content PCa tissues.^18^

### 2.3 Multiplex immunohistochemistry

mIHC was performed as described by Blom et al.^17^ See full details in Supplementary Information. Briefly, for a 5‐plex staining, paraffin was removed from the FFPE and heat‐induced epitope retrieval (HIER) was performed. After HIER, endogenous peroxide activity and protein blocking was performed and the first and second primary antibodies were detected using tyramide signal amplification for AlexaFluor488 and AlexaFluor555, respectively (PerkinElmer, Waltham, MA). The third and fourth primary antibodies raised in different species were detected using AlexaFluor647 and AlexaFluor750 fluorochrome‐conjugated secondary antibodies. Nuclei were counterstained using 4′,6‐diamidino‐2‐phenylindole (DAPI).

### 2.4 Image analysis

**Figure 1:**
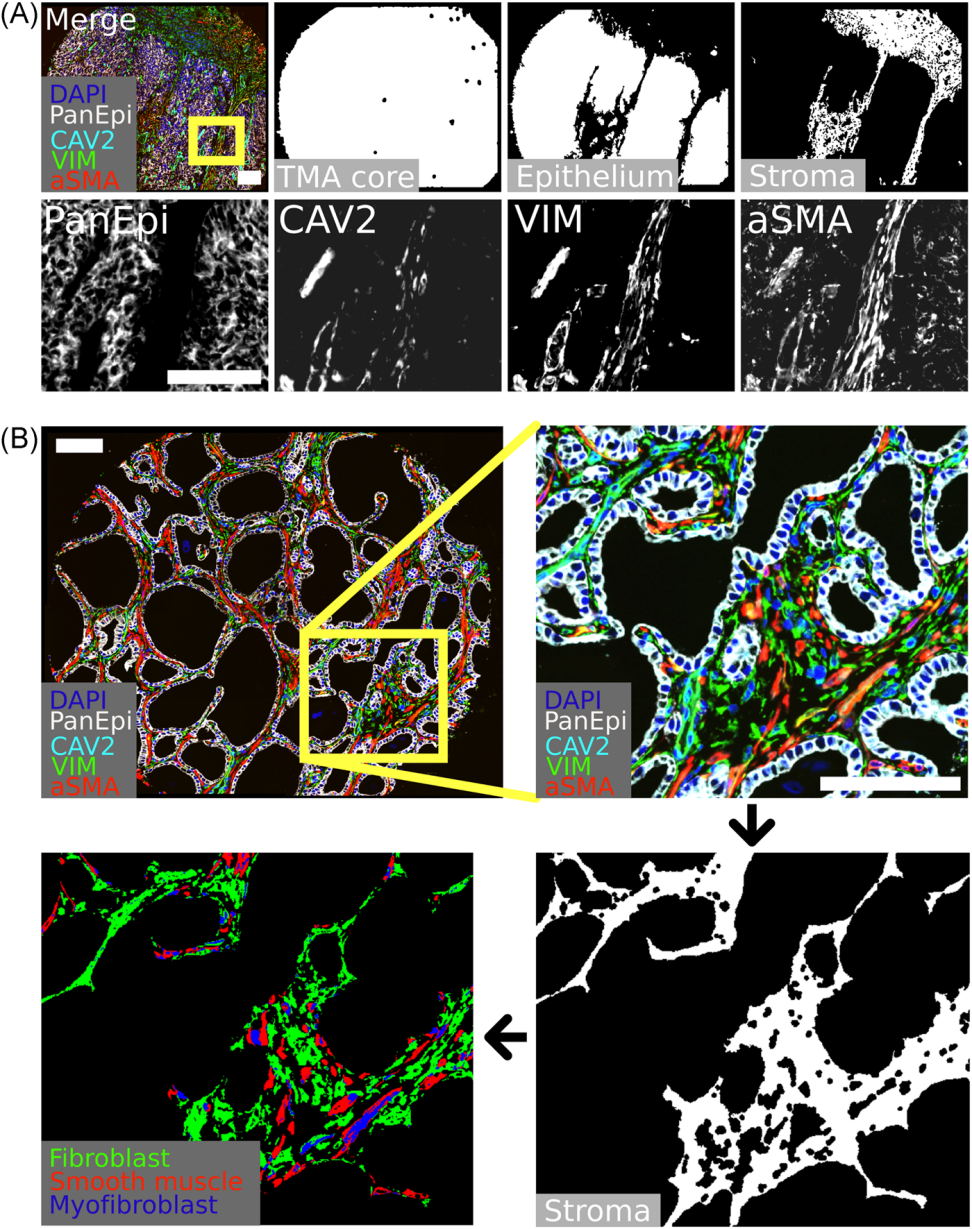
Multiplexed immunohistochemistry (mIHC) and stromal cell classification. A, Representative example of mIHC‐stained TMA core for indicated markers and compartment segmentation. B, Representative example for stromal segmentation and stromal cell classification. Scale bar 100 µm. aSMA, alpha‐smooth muscle actin; CAV2, Caveolin‐2; DAPI, 4′,6‐diamidino‐2‐phenylindole; VIM, vimentin [Color figure can be viewed at wileyonlinelibrary.com]

All image analyses were performed using CellProfiler^19^ (version 2.2.0) (see Supplementary Information and Supplementary Data for details). The threshold for PanEpi channel was set manually to exclude all of the stromal area based on visual inspection. The PanEpi detection included staining and detection of two different anti‐pan‐cytokeratin antibody clones (AE1/3 and C‐11) and anti‐E‐cadherin antibody (clone 36) for optimal epithelium coverage.^20^ Stromal cells were classified as fibroblasts (VIM‐pos, aSMA‐neg), myofibroblasts (VIM‐pos, aSMA‐pos), or smooth muscle (aSMA‐pos, VIM‐neg) (Figure 1; Figure S1).

The intensity of each stained marker was normalized across TMA cores of all patients. The average of the normalized marker intensity per patient was used as the final metric. The relative area for each stromal cell class (fibroblast, myofibroblast, and smooth muscle) was measured as the area of positive pixels or cell counts within stroma in each TMA core. For a given cell class, the average relative area or relative cell count in the stroma in all TMA cores per patient was used as the final metric. Cell class variables were categorized in quartiles when appropriate.

### 2.5 Statistical analysis

Demographics for patients with high‐quality image data are presented in Table S2. Patients with missing clinicopathological data were excluded from final analyses where the missing data were considered relevant. For example, in multivariate survival analyses, only patients with complete data for all the variables were included.

The Mann‐Whitney U test was used to test differences between two groups of non‐normally distributed continuous or categorial variables, and *t* test for normally distributed continuous variables. An independent Kruskal‐Wallis H test with pairwise Dunn's post hoc test was used to test differences between three or more non‐normally distributed continuous or categorical variable groups. Normality of data was tested using Shapiro–Wilk tests. Correlations between continuous variables were calculated using the two‐tailed Pearson product moment correlation coefficient function, and *P* value using two‐tailed Student *t* test. Unsupervised hierarchical clustering and data plotting were performed using ClustVis^21^ online tool.

For survival analyses, we used Cox proportional hazard regression model and Kaplan‐Meier plots with the Wald test and log‐rank, respectively. Proportional hazard assumption was tested for given variables using the Schoenfeld test. The percentage proportions of the stromal cell classes were multiplied by 10 for the Cox proportional hazard regression in order to yield hazard ratios for a ten percent change in the proportion.

If multiple tests were performed, *P* values were controlled for 20% false discovery rate using the Benjamini‐Hochberg step‐up procedure.^22^ *P* < .05 were considered statistically significant. All statistical analyses and data plotting were performed using the IBM SPSS 24 (SPSS Inc, Armonk, NY).

### 2.6 Additional information

Ethical approval and consent to participate: Ethical approval for the use of tissue material and clinicopathological data was obtained from the Institutional Ethics Committee of Hospital District of Helsinki and Uusimaa (D:no 446/13/03/02/2009) and from the National Institute for Health and Welfare (D:no THL490.5.05.00/2016) according to the national legislation. A permission for retrospective use of patient data and the archived tissue blocks was approved by the National Supervisory Authority for Welfare and Health (VALVIRA, D:no 4076/32/300/02).

## 3 RESULTS

### 3.1 Elevated expression of stromal vimentin is associated with aggressive PCa

**Figure 2:**
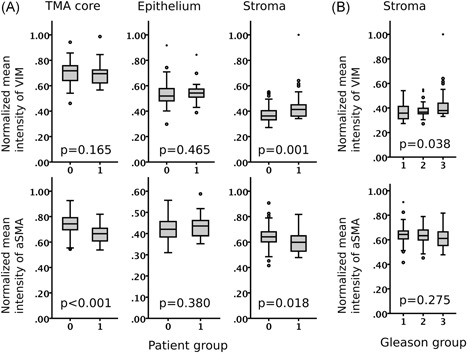
High‐risk prostate cancer shows increased expression of stromal vimentin (VIM) and reduced expression of stromal alpha‐smooth muscle actin (aSMA). A, Vimentin (VIM) and alpha‐smooth muscle actin (aSMA) expression (mean intensity) in full TMA cores, epithelium, and stroma of prostate cancer patients without cancer‐specific event (0) and with cancer‐specific event (1). Mann‐Whitney U test. B, Stromal expression of VIM and aSMA in tumors with Gleason score <7 (1), 7, (2), and >7 (3). Boxplot indicates minimum, first quartile, median, third quartile, and maximum. Individual data points indicate outliers (circle and asterisk). Kruskal‐Wallis H test. Cohort I (n = 115)

In Cohort I, 117 patients (74%) had high‐quality image data. We systematically assessed VIM and aSMA expression in full TMA cores or in the epithelium and the stromal compartment of each core. Representative IHC staining examples of VIM and aSMA in PCa are provided as supplementary data (Figure S2). VIM expression was significantly higher (*P* < .001) and aSMA lower (*P* = .018) in the stroma of patients dying of PCa compared with those of who did not die during the follow‐up time (Figure 2A). Further, stromal VIM expression was higher in high‐grade tumors whereas stromal aSMA expression was not significantly different in the different grade groups (Figure 2B).

### 3.2 Enriched fibroblasts and reduced smooth muscle in prostate cancer stroma associate with adverse clinical outcome

**Figure 3:**
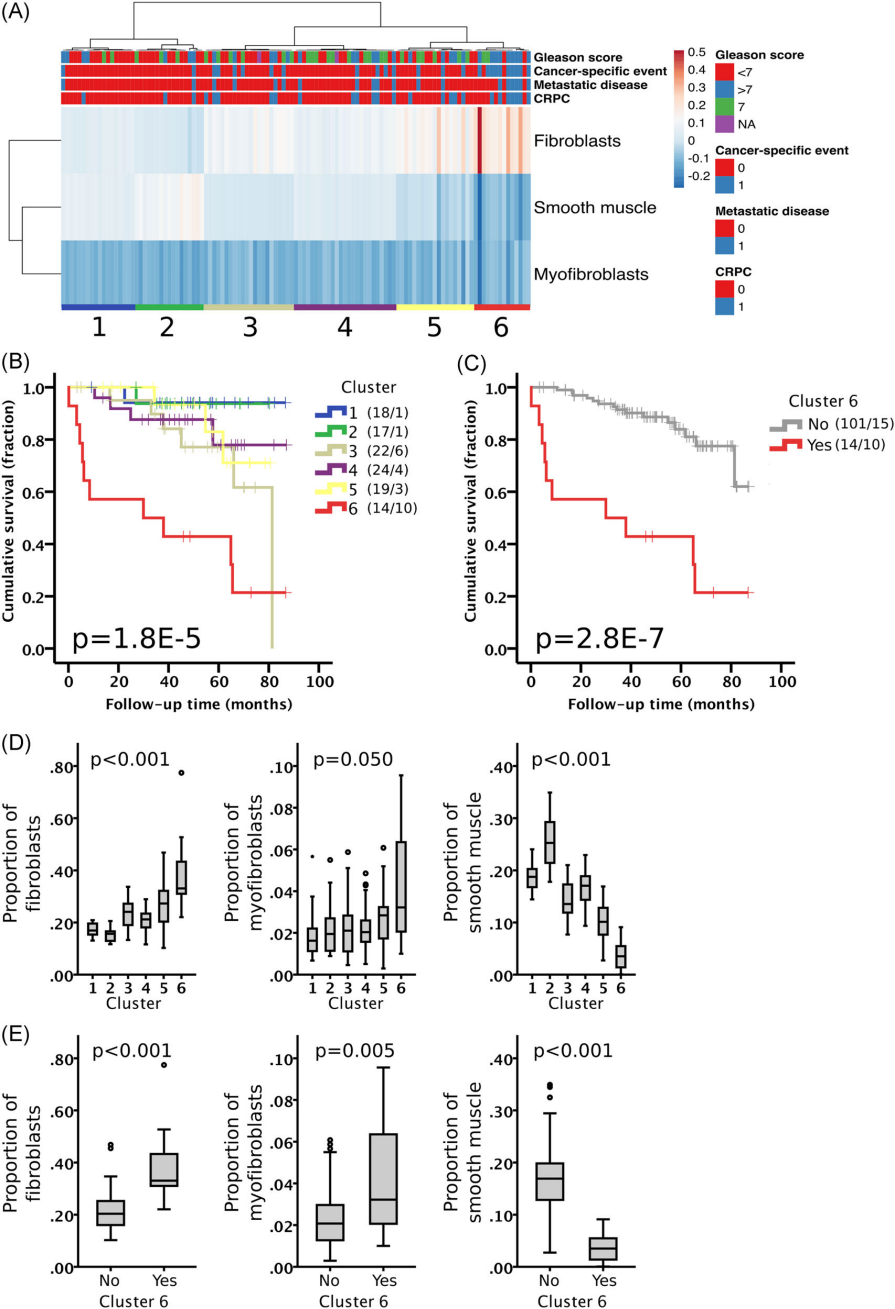
Stroma of aggressive prostate cancer is enriched in fibroblasts and deprived of smooth muscle. (A) Unsupervised hierarchical clustering of patients based on the relative proportion of each cell type in stroma in Cohort I (n = 115). Columns are clustered using Euclidean distance and average linkage. Rows are clustered using correlation distance and average linkage. Cluster of patients is indicated with a number (1‐6). Heatmap color indicates scaled proportion of cells in stroma (arbitrary units). CRPC, castration‐resistant prostate cancer. (B) Kaplan‐Meier analysis for cancer‐specific mortality of the different clusters separately and (C) Clusters 1‐5 (grey) vs. Cluster 6 (red). Number of patients and events are presented in brackets. Log‐rank test. (D) Proportion of the different stromal cells in the different clusters (1‐6). Kruskal‐Wallis H test. (E) Proportion of the different stromal cells in Cluster 1‐5 vs Cluster 6. Mann‐Whitney U test. Boxplot indicates minimum, first quartile, median, third quartile, and maximum. Individual data points (circle and asterisk) are indicating outliers [Color figure can be viewed at wileyonlinelibrary.com]

The association of the stromal expression of VIM and aSMA with aggressive cancer led us to further classify the different stromal cells as fibroblasts (VIM‐positive and aSMA‐negative), myofibroblasts (VIM‐pos and aSMA‐pos), and smooth muscle cells (aSMA‐pos and VIM‐neg). Unsupervised hierarchical clustering of these stromal cell subtypes resulted in six major phenotype clusters (Figure 3A). Tumors with high grade, metastatic disease (MET), and castration‐resistant prostate cancer (CRPC) were significantly enriched in phenotype Cluster 6 (n = 14 patients) as compared to phenotype Clusters 1‐5 (n = 101 patients) (Χ^2^; Grade, *P* = .006; MET, *P* = .005; CRPC, *P* = .009). Kaplan‐Meier analysis showed that patients in Cluster 6 had very poor cancer‐specific outcome compared with patients in Clusters 1‐5 (*P* = 2.8E‐7; Figure 3B,C). The proportion of fibroblasts and myofibroblasts was higher and the proportion of smooth muscle cells lower in Cluster 6 compared with other clusters (Figure 3D). Specifically, patients in Cluster 6 had 83% more fibroblasts but 77% less smooth muscle than in Clusters 1‐5. (Figure 3E).

### 3.3 The proportion of fibroblasts in stroma is an independent predictor of clinical outcome

**Figure 4:**
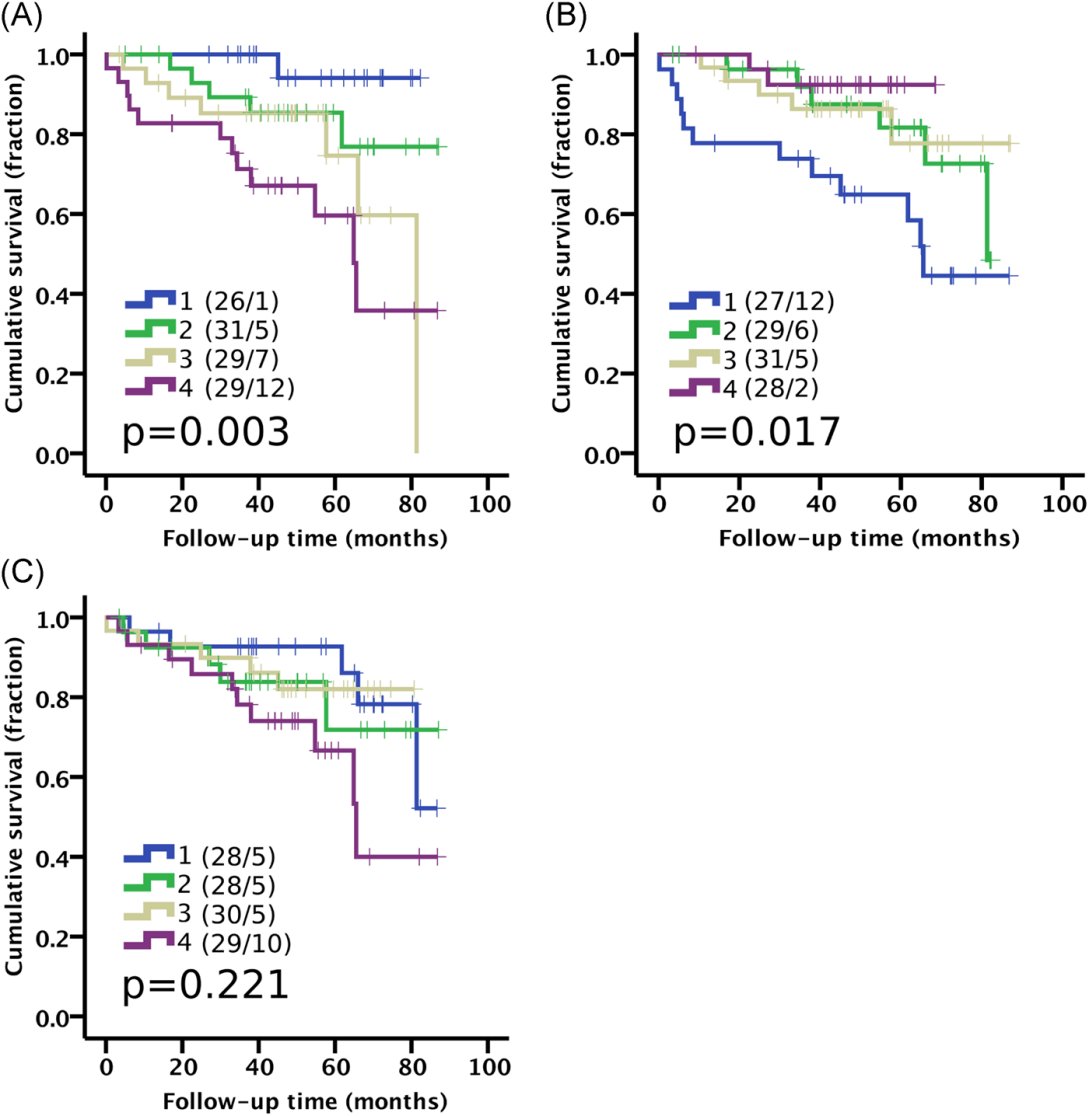
High proportion of fibroblasts and low proportion of smooth muscle cells predicts prostate cancer‐specific mortality. Kaplan‐Meier analysis using quartile categories (1‐4) for the proportion of stromal (A) fibroblast, (B) smooth muscle, and (C) myofibroblasts. Number of patients and events are presented in brackets. Log‐rank test. Cohort I (n = 115) [Color figure can be viewed at wileyonlinelibrary.com]

As the “fibroblast‐high, smooth muscle‐low” phenotype of Cluster 6 showed clear association with poor survival and aggressive cancer, we then studied how individual cell types predict outcome. Kaplan‐Meier analysis and univariate Cox regression analysis showed that both having a high proportion of fibroblasts (HR 2.36; *P* = 9.23E‐7) or a low proportion of smooth muscle cells hazard ratio (HR 0.39; *P* = .002) separately predicted poor clinical outcome whereas the proportion of myofibroblasts was not predictive of outcome (HR 5.45; *P* = .061) (Figure 4; Table 1). Importantly, fibroblasts predicted cancer‐specific outcome (PCSM) in a multivariate Cox regression analysis when adjusted for Gleason score and age at diagnosis (HR 1.88; *P* = .001). Further, we validated the prognostic effect of fibroblast counts by using an alternative image analysis method based on digital stromal cell detection by nuclear segmentation (Figure S3). Moreover, the proportion of fibroblasts, smooth muscle, or myofibroblasts was not significantly different in tumors with different grade (Figure S4), although high‐grade tumors had significantly less stroma compared with low‐grade tumors (*P* < .001). We observed an inverse association between the proportion of fibroblast and the total stromal area, whereas the proportion of smooth muscle cells weakly correlated with the total stromal area (Figure S5).

**Table 1:** Hazard ratios (HR) in univariate and multivariate Cox Proportional Hazard regression analysis for prostate cancer‐specific mortality (PCSM) in Cohort I and Cohort II. HR for the different cell classes is reported as per 10% change in the proportion of the indicated cell class. Dx, diagnosis, Tx, treatment. Bolded *P* values remain significant after Benjamini‐Hochberg procedure. Wald test

	Univariate HR (CI, 95%)	*P*	n	Multivariate HR (CI, 95%)	*P*	n
Cohort I						113
Fibroblasts	2.36 (1.68‐3.33)	**9.23E‐07**	115	1.88 (1.30‐2.71)	**.001**	
Smooth muscle	0.39 (0.21‐0.71)	**.002**	115	–	–	
Myofibroblasts	5.45 (0.92‐32.2)	.061	115	–	–	
Gleason score						
<7	0 (0‐3.3E85)	.899	54	2E‐6 (0‐1.3E88)	.905	
7	0.15 (0.55‐0.42)	**.00029**	29	0.18 (0.06‐0.53)	**.002**	
>7	1	**.0003**	32	1	**.008**	
Age at Dx	1.12 (1.07‐1.18)	**.00003**	117	1.04 (0.99‐1.09)	.136	
Cohort II						240
Fibroblasts	1.89 (1.17‐3.01)	**.009**	248	1.73 (1.04‐2.86)	**.034**	
Smooth muscle	0.68 (0.50‐1.00)	.051	248	–	–	
Myofibroblasts	0.82 (0.54‐1.25)	.349	248	–	–	
Gleason score						
<7	0.15 (0.03‐0.70)	**.016**	64	0.21 (0.04‐1.04)	.056	
7	0.49 (0.20‐1.17)	.108	159	0.68 (0.27‐1.74)	.423	
>7	1	**.042**	37	1	.161	
Age at Tx	1.04 (0.97‐1.11)	.239	269	1.03 (0.96‐1‐11)	.386	

Abbreviation: CI, confidence interval

Bolded *P* values remain significant after Benjamini‐Hochberg procedure (*P* < 0.05).

**Figure 5:**
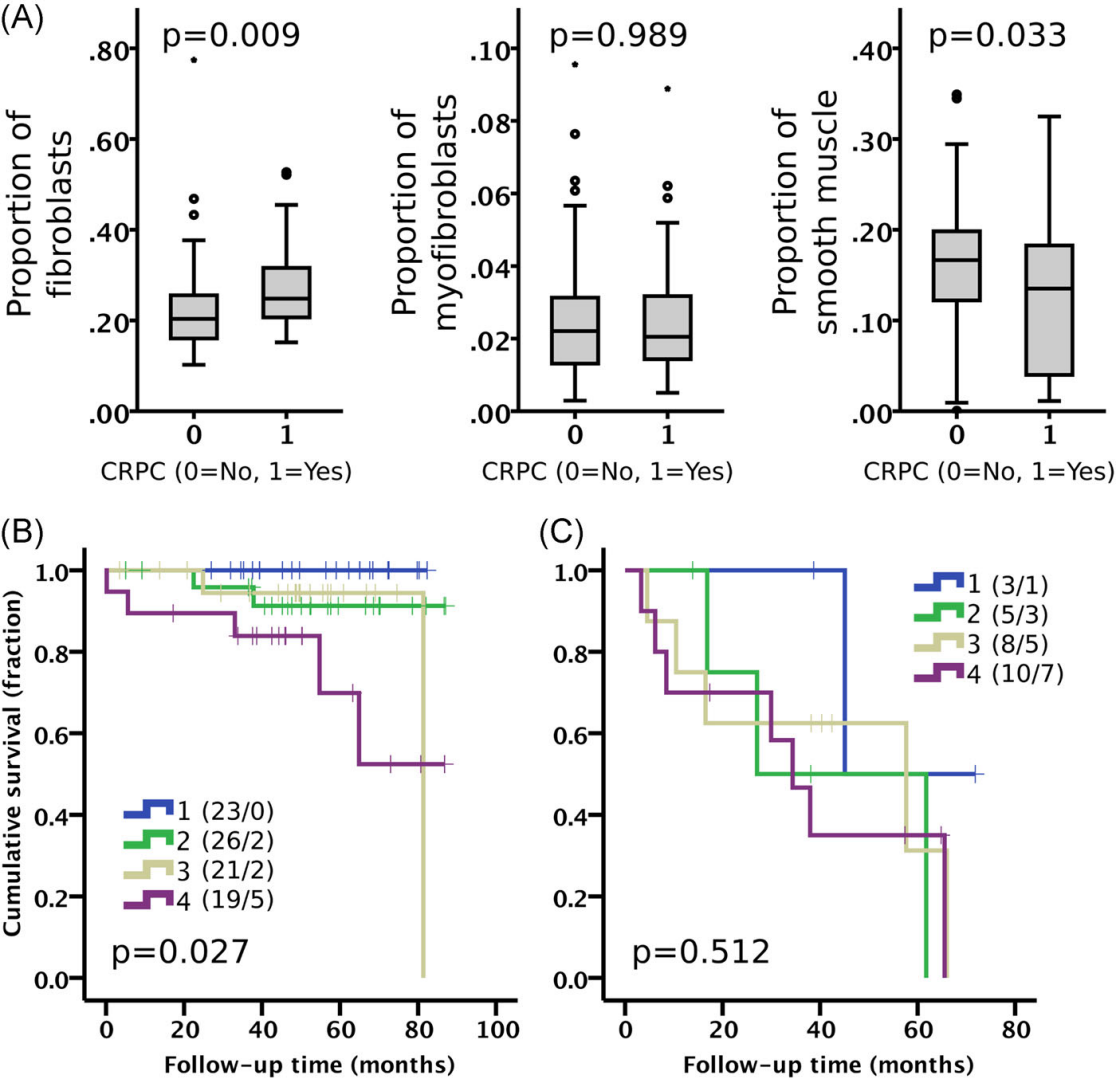
(A) The proportion of stromal cell types in non‐CRPC (0, n = 89) and in CRPC (1, n = 26) cases. Mann‐Whitney U test. Boxplot indicates minimum, first quartile, median, third quartile, and maximum. Individual data points (circle and asterisk) are indicating outliers. Kaplan‐Meier analysis of proportion of fibroblasts (quartiles 1‐4) stratified in (B) non‐CRPC and (C) CRPC cases. Number of patients and events are presented in brackets. Log‐rank test. Cohort I. CRPC, castration‐resistant prostate cancer [Color figure can be viewed at wileyonlinelibrary.com]

As Cluster 6 was enriched in CRPC, we studied the non‐CRPC and CRPC patients separately. The proportions of fibroblasts were significantly higher and those of smooth muscle cells lower in CRPC cases compared with non‐CRPC cases (Figure 5A). Despite the difference, the proportion of fibroblasts predicted cancer‐specific patient survival also in non‐CRPC cases in Kaplan‐Meier analysis (*P* = .027) and in Cox regression univariate (HR 2.28; *P* = .001) and multivariate (HR 2.18; *P* = .037) analyses (Figure 5B; Table S3).

### 3.4 Fibroblasts predict cancer‐specific outcome in an independent prostatectomy cohort

The prognostic effect of fibroblasts was validated in Cohort II which comprised radical prostatectomy samples from primary PCa. In Cohort II, 340 patients (97%) had high‐quality image data and the distribution of the proportion of fibroblasts was similar to that of Cohort I (Figure S6). Univariate Cox regression analysis of the proportion of fibroblasts in the stroma showed a significantly worse cancer‐specific outcome with increasing proportion of fibroblasts (HR 1.89; *P* = .009; Table 1). Moreover, a high proportion of fibroblasts was validated as an independent predictor of PCSM in a multivariate Cox regression analysis when adjusted for Gleason score and age at prostatectomy (HR 1.73; *P* = .034). Interestingly, the proportion of fibroblasts in matched benign cores (from the same patients adjacent to tumor) also predicted PCSM when comparing the highest and lowest quartiles in Kaplan‐Meier analysis (Figure S7). However, the Cox regression analysis did not reach statistical significance (HR 1.51; *P* = .093) for the proportion of fibroblasts in the benign TMA cores. Benign cores were available only in Cohort II. We found no significant associations between the proportion of fibroblasts and the expression of AR or PTEN proteins (Figure S8).

Although VIM is also expressed in endothelium and in immune cells, we ensured the correct classification of fibroblasts by excluding VIM‐positive endothelium using CAV2 signal (strongly expressed in endothelium). The average difference between fibroblast count before and after endothelium exclusion was negligible (0.4%, n = 115). The effect of immune cells was controlled by visually assessing the amount of stromal VIM‐pos immune cells in IHC‐stained sections in Cohort II. We found no difference in the proportion of fibroblasts between patients with different amounts of stromal immune cells (Figure S9). Together, the effect of endothelium and immune cells on the proportion of fibroblasts was small.

## 4 DISCUSSION

We quantified the cell subtype composition of human PCa stroma by using mIHC, as well as systematic and quantitative digital image analysis. To our knowledge, this is the first quantitative study showing that a high proportion of IHC‐classified fibroblasts predicts poor cancer‐specific outcome. A high proportion of fibroblasts was also associated with CRPC. Tuxhorn et al^23^ have shown that VIM‐positive cells are enriched in early PCa and in prostate intraepithelial neoplasia and Ayala et al^11^ showed that collagen‐rich stroma and a low proportion of smooth muscle is predictive for biochemical recurrence in PCa. However, in both studies, stroma was analyzed with unspecific stains or single‐colour IHC. Further, semi‐quantitative visual analyses of the highly variable PCa stroma^11^, ^24^, ^25^ are error‐prone^26^ and do not allow high‐resolution, quantitative analysis of the stromal cell compartments.

Our data expand beyond previous evidence suggesting the clinical significance of stroma in PCa. Our results of fibroblast‐rich stroma predicting poor survival in hormone‐naive PCa is in line with Mo et al^13^ who showed that high *VIM* gene expression predicts metastatic potential in primary PCa. Moreover, we show that fibroblast‐rich stroma associated with CRPC, an observation supported by Thalmann et al^27^ who showed that human cancer‐associated fibroblasts may confer castration resistance of LNCaP cell line in vivo, suggesting that fibroblasts may have an active role in the development of CRPC. Further, we observed that fibroblasts in the benign stroma (adjacent to cancer) associate with poor outcome, supporting earlier studies where tumor‐associated stromal features were shown to be present in nonmalignant stroma of prostate^28^ and breast^29^ tissues. In contrast, the reduction seen in the amount of smooth muscle cells in aggressive PCa is attributable to the fact that high‐grade PCa tumors have less stroma in general, supported by our data showing that smooth muscle is positively correlated with the amount of stroma present in the tumors and that high‐grade tumors have less stroma.

Together, these data suggest that fibroblast‐rich stroma is not only a reaction to the tumor but is an active component in the tumor development. Quantitation of fibroblasts should, therefore, be considered in developing preventive, diagnostic, predictive, and prognostic tests for prostate cancer. Our data indicate that fibroblasts are strongly associated with aggressive PCa, therefore being a potential target for the development of therapeutic approaches.

mIHC and automatic, unbiased image analysis is a powerful tool to study solid tumors, allowing quantitative studies of single cells in situ and excluding observer biases inherent in previous studies. To test the robustness of our cell classification, we assessed the effect of immune cells and endothelium in fibroblast classification. We observed a low proportion of immune cells in PCa stroma, which is in agreement with the RNA expression data from The Cancer Genome Atlas.^24^ Together, our controls show that the effect of immune cells or endothelium in the fibroblast analysis is likely to be negligible.

While both clinical cohorts have many cancer‐specific events, the cohorts are different in terms of clinical disease profile as well as sample collection period. As a limitation, both cohorts are observational retrospective cohorts, where therapy after TURP or prostatectomy was not pre‐determined and therefore may introduce confounding bias in patient survival. However, the fact that the same conclusions were obtained from both cohorts suggest that our key observations are robust and validated. Importantly, the fibroblast‐rich stroma was prognostic in both cohorts independent of tumor grade and patient age in a Cox regression model.

## 5 CONCLUSION

Comprehensive multiplexed mIHC and quantitative digital image analysis facilitates high‐resolution and unbiased analysis of the cell subtypes in the cancer‐associated stroma in human PCa. We conclude that a fibroblast‐rich stroma associates with aggressive disease and adverse clinical outcome. The study provides evidence for the clinical significance of fibroblast‐rich stroma in human PCa that could have implications for prevention, diagnosis, prognostication and therapy of prostate cancer.

## CONFLICT OF INTERESTS

Sami Blom is an employee of Aiforia Technologies Oy, a company providing image analysis products and services. Other authors declare no conflict of interests.

## Supporting information

Supporting information
