# Supplementary material for: Fibroblast as a critical stromal cell type determining prognosis in prostate cancer
Source: Prostate. 2019 Jul 3;79(13):1505–13. doi: 10.1002/pros.23867 (PMC6813917; doi:10.1002/pros.23867)
Supplement: Supplementary file 1 — Supporting information [file PROS-79-1505-s001.doc]

**Supplementary Information for “Fibroblast as a critical stromal cell type determining prognosis in prostate cancer”**

Sami Blom^1^, Andrew Erickson^1^, Arne Östman^2^, Antti Rannikko^3^, Tuomas Mirtti^1,3^, Olli Kallioniemi^1,2^, Teijo Pellinen^1^

^1^Institute for Molecular Medicine Finland (FIMM), Helsinki Institute of Life Science (HiLIFE), University of Helsinki, Helsinki, Finland

^2^Science for Life Laboratory, Department of Oncology & Pathology, Karolinska Institutet, Stockholm, Sweden

^3^Urology and Pathology, Helsinki University Hospital, Helsinki, Finland

**Corresponding author:**

Dr Teijo Pellinen,

Institute for Molecular Medicine Finland (FIMM)

Biomedicum Helsinki 2U

P.O. Box 20 (Tukholmankatu 8)

FI-00014 University of Helsinki, Finland

**Supplementary Materials and Methods**

**Patient material**

Study cohorts were formalin-fixed paraffin embedded (FFPE) samples from transurethral resection of prostate (TURP; Cohort I, n=159 patients) and retrospective radical prostatectomy (Cohort II, n=435 patients). Tissue microarrays (TMA) were constructed for Cohort I by taking 1-5 cores per patient and for Cohort II as described earlier^1^. In Cohort I, the TMA cores were selected from histologically confirmed carcinoma areas in the TURP chips, and the number of cores depended on the quantity of chips available. Prostate cancer-specific mortality (PCSM) was recorded as end-point in both cohorts. Tumour grading was based on Gleason grading system^2^, and was performed by a board certified pathologist specialized in urothelial cancers (T.M.). Castration-resistant prostate cancer (CRPC) was defined by rising PSA levels or radiological progression during anti-androgen treatment. Age at diagnosis and age at prostatectomy were recorded for Cohort I and Cohort II, respectively. Cohort II included only patients who had non-metastatic (M0) primary PCa at diagnosis and who received prostatectomy as the first treatment (excluding also neo-adjuvant therapy). Phosphatase and tensin homolog (PTEN) and androgen receptor (AR) protein expression data were obtained from Lahdensuo et al.^3^ and Sahu et al.^1^, respectively, for Cohort II.

Ethical approval for the use of tissue material and clinicopathological data was obtained from the Institutional Ethics Committee of Hospital District of Helsinki and Uusimaa (D:no 446/13/03/02/2009) and from the National Institute for Health and Welfare (D:no THL490.5.05.00/2016) according to the national legislation. A permission for retrospective use of patient data and the archived tissue blocks was approved by the National Supervisory Authority for Welfare and Health (VALVIRA, D:no 4076/32/300/02).

**Immunohistochemistry**

Antibodies used in the study are listed in Supplementary Table S1. Antibodies for VIM, aSMA, and CAV2 were optimized for mIHC using immunohistochemistry in prostatectomy samples as described earlier^4^. Antibodies for PanEpi (PanCK (clones AE3/1 and C11) + E-cadherin (clone 36) were tested earlier for mIHC^4^. Digital, whole-slide brightfield images of the IHC-stained sections were acquired at 0.22 µm/pixel resolution using Pannoramic P250 Flash II whole-slide scanner (3DHistech, Hungary) equipped with Zeiss Plan-Apochromat 20x objective (NA 0.8). After image acquisition, brightfield images were converted to JPEG2000 format (95% quality) and subsequently into virtual slides compressed to a wavelet file format (Enhanced Compressed Wavelet, ECW, ER Mapper, Erdas Inc, Atlanta, Georgia) with 1:5 compression ratio. Virtual slides were uploaded to WebMicroscope platform (Aiforia Technologies Oy, Finland) for visual scoring. The amount of VIM-positive immune cells per patient was visually scored by S.B. in Cohort II as “low”, “medium”, and “high” if <1%, 1–5%, or >5% of stromal area was occupied by VIM-positive immune cells, respectively.

**Multiplex immunohistochemistry**

Multiplex immunohistochemistry (mIHC) was performed as described by Blom et al.^4^. Briefly, for a 5-plex staining, paraffin was removed from the FFPE and heat-induced epitope retrieval (HIER) was performed. After HIER, endogenous peroxide activity and protein blocking was performed in 0.9% H_2_O_2_ and in 10% normal goat serum, respectively. After protein blocking, we applied the first primary antibody and a species-specific HRP-conjugated secondary antibody. Tyramide signal amplification (TSA) for AlexaFluor488 (PerkinElmer, Waltham, MA) was applied on the slides according to manufacturer´s instructions and the reaction was incubated for exactly 15 min. HIER was performed to denature the antibody complex and to attenuate the enzymatic activity of HRP. The TSA reaction was repeated for a second primary antibody using AlexaFluor555. After second HIER, a pair of primary antibodies raised in different species was used to detect additional two targets and were detected using AlexaFluor647 and AlexaFluor750 fluorochrome-conjugated secondary antibodies. Nuclei were counterstained using DAPI and slides were mounted and coverslips applied. Digital, whole-slide fluorescence images of mIHC slides were acquired at 0.32 µm/pixel resolution using Axio Imager.Z2 microscope (Zeiss, Germany) equipped with EC Plan-Neofluar 20x objective (NA 0.8), Metafer scanning platform with CoolCube 2 CCD camera (MetaSystems, Germany), PhotoFluor LM-75 metal halide light source (89 North, Williston VT), and DAPI, FITC, CY3, CY5, and CY7 filter sets. After image acquisition, images were converted to 8-bit JPEG2000 format (95% quality).

**Image analysis**

All image analyses were performed using CellProfiler^5^ (version 2.20). The segmentation mask of each TMA core was calculated using CellProfiler´s ‘*maximum’* command across all fluorescence channels (VIM+aSMA+CAV2+PanEpi+nuclei) yielding binary image(TMA core). Epithelium mask was segmented using binary image(PanEpi) within binary image(TMA core). The threshold for PanEpi channel was set manually to exclude all stromal area based on visual inspection. The PanEpi detection included staining and detection of two different anti-pan-cytokeratin antibody clones (AE1/3 and C-11) and anti-E-cadherin antibody (clone 36) for optimal epithelium coverage. For stromal segmentation, we first computed a binary image(VIM+aSMA+CAV2). The stromal mask was defined as a binary image(VIM+aSMA+CAV2)–binary image(PanEpi) within binary image(TMA core) resulting in binary image(stroma). Pixels within the binary image(stroma) were classified to belong to either fibroblasts (VIM-pos, aSMA-neg), myofibroblasts (VIM-pos, aSMA-pos), or smooth muscle (aSMA-pos, VIM-neg). The threshold values for VIM and aSMA positivity were set manually so that smooth muscle cells were negative for VIM and immune cells and endothelial cells were negative for aSMA. We set thresholds independently in the patient cohorts. For digital cell counting, nuclei were first segmented using adaptive Otsu thresholding^6^. Nuclei with >50% overlap with stromal binary image were considered stromal nuclei. The mean intensity of the highest 25% of pixels within the periphery (10-pixel expansion) of each stromal nucleus were used to classify the cells as above.

The intensity of each stained marker was normalized across TMA cores of all patients. The average of the normalized marker intensity per patient was used as the final metric. The relative area for each stromal cell class (fibroblast, myofibroblast, and smooth muscle) was measured as the area of positive pixels or a fraction of cells from all cells within stroma in each TMA core. For a given cell class, the average relative area or cell count in all TMA cores per patient was used as the final metric. Cell class variables were categorized in quartiles if needed.

We assessed the quality of TMA cores by visual inspection. Any TMA cores with low quality (e.g. ruptured or folded tissue or staining artefact) were excluded from further analyses. In both cohorts, only patients with at least two high-quality TMA cores were included in final analyses except if the patient had only a single TMA core available, in which case the patient was included in the final analyses if the single core was of high-quality.

**Supplementary References**

1. Sahu B, Laakso M, Ovaska K, et al. Dual role of FoxA1 in androgen receptor binding to chromatin, androgen signalling and prostate cancer. *The EMBO journal.* 2011;30(19):3962-3976.

2. Epstein JI, Allsbrook WC, Jr., Amin MB, Egevad LL, Committee IG. The 2005 International Society of Urological Pathology (ISUP) Consensus Conference on Gleason Grading of Prostatic Carcinoma. *Am J Surg Pathol.* 2005;29(9):1228-1242.

3. Lahdensuo K, Erickson A, Saarinen I, et al. Loss of PTEN expression in ERG-negative prostate cancer predicts secondary therapies and leads to shorter disease-specific survival time after radical prostatectomy. *Mod Pathol.* 2016;29(12):1565-1574.

4. Blom S, Paavolainen L, Bychkov D, et al. Systems pathology by multiplexed immunohistochemistry and whole-slide digital image analysis. *Sci Rep.* 2017;7(1):15580.

5. Carpenter AE, Jones TR, Lamprecht MR, et al. CellProfiler: image analysis software for identifying and quantifying cell phenotypes. *Genome Biol.* 2006;7(10):R100.

6. Otsu N. A Threshold Selection Method from Gray-Level Histograms. *IEEE Transactions on Systems, Man, and Cybernetics.* 1979;9(1):62-66.

**Supplementary Figures and Tables**


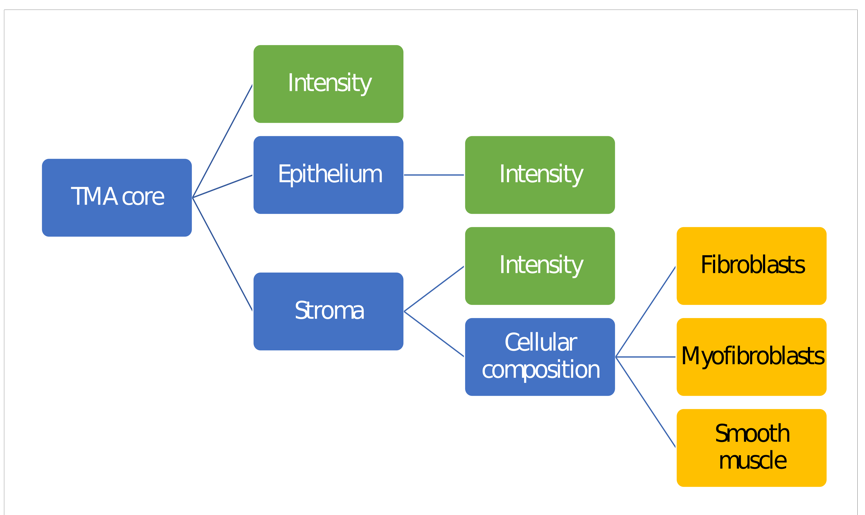


Figure S1. Image analysis scheme in the study.


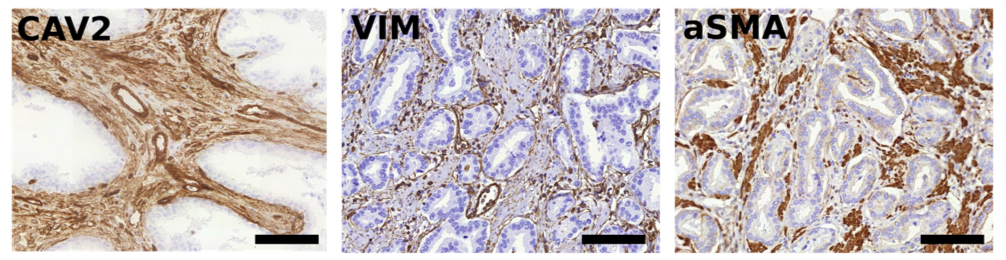


Figure S2. Representative images for Caveolin-2 (CAV2), Vimentin (VIM), and alpha-smooth muscle actin (aSMA) immunohistochemistry in prostatectomy samples. Scale bar 100 µm.


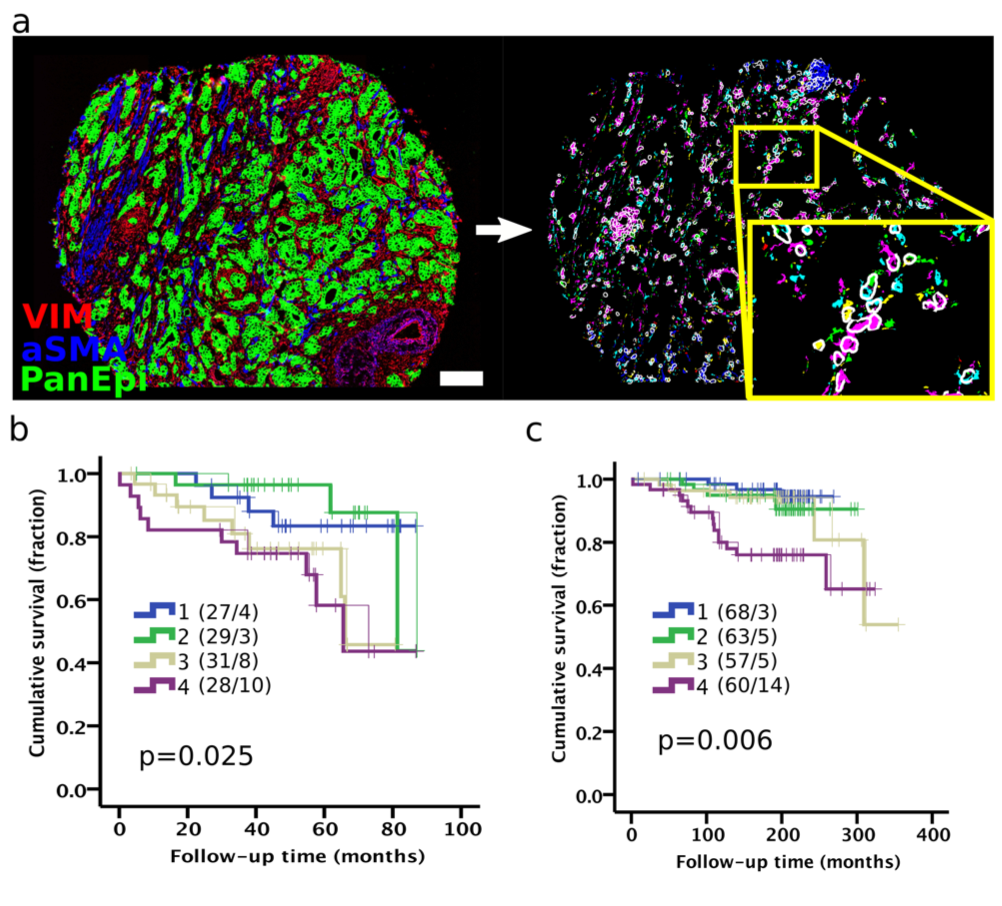


Figure S3. Digital fibroblast cell counting based on nuclear segmentation validates the results of area-based image analysis. (a) Representative example of a mIHC-stained TMA core analysed using both area-based (right panel: coloured pixels) and nuclear segmentation based (right panel: white contours) image analysis. Scale bar 100 µm. Kaplan-Meier analysis in (b) Cohort I (n=115) and in (c) Cohort II (n=248) using quartile categories (1–4) for the proportion of stromal fibroblast from all stromal cells (digital counting). Number of patients and events are presented in brackets. Log-rank test.


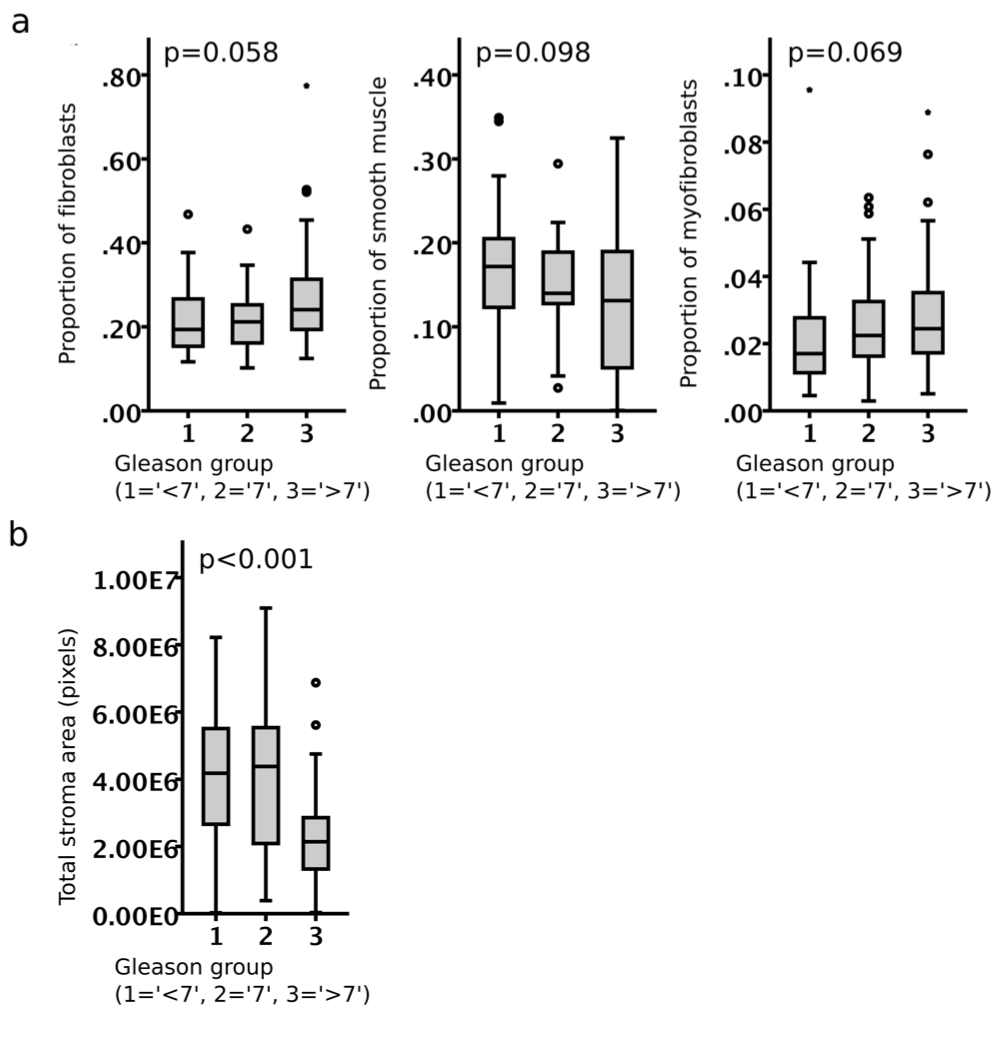


Figure S4. Distribution of (a) fibroblasts, smooth muscle cells, and myofibroblasts (n=113) as well as (b) the total stromal area (n=115) in tumours with different grade (Gleason score). Kruskal-Wallis H test. Boxplot indicates minimum, first quartile, median, third quartile, and maximum. Individual data points (circle and asterisk) are indicating outliers.


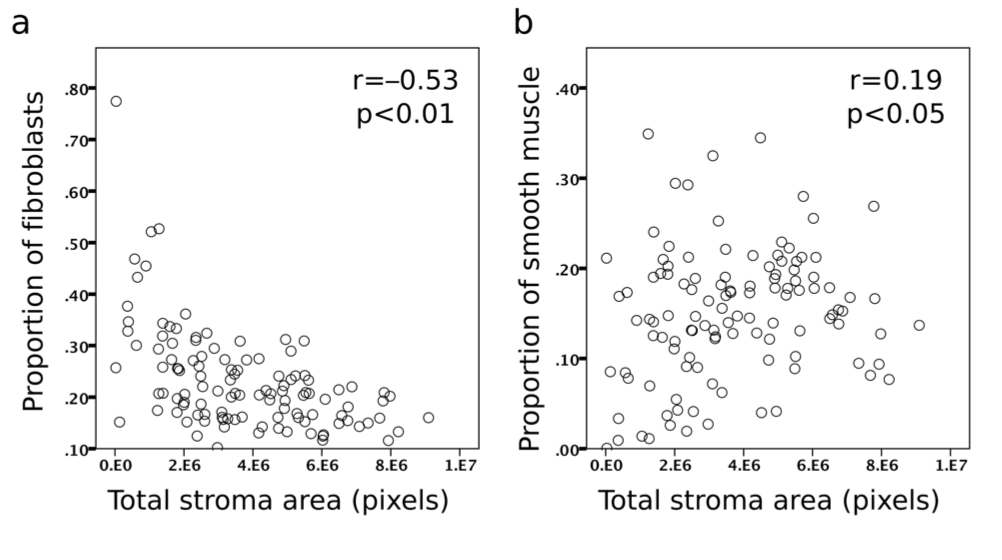


Figure S5. Correlation of proportion of total stromal area with (a) fibroblasts (n=117) and (b) smooth muscle (n=115) in Cohort I. r, Pearson correlation coefficient; two-tailed Student´s t-test.


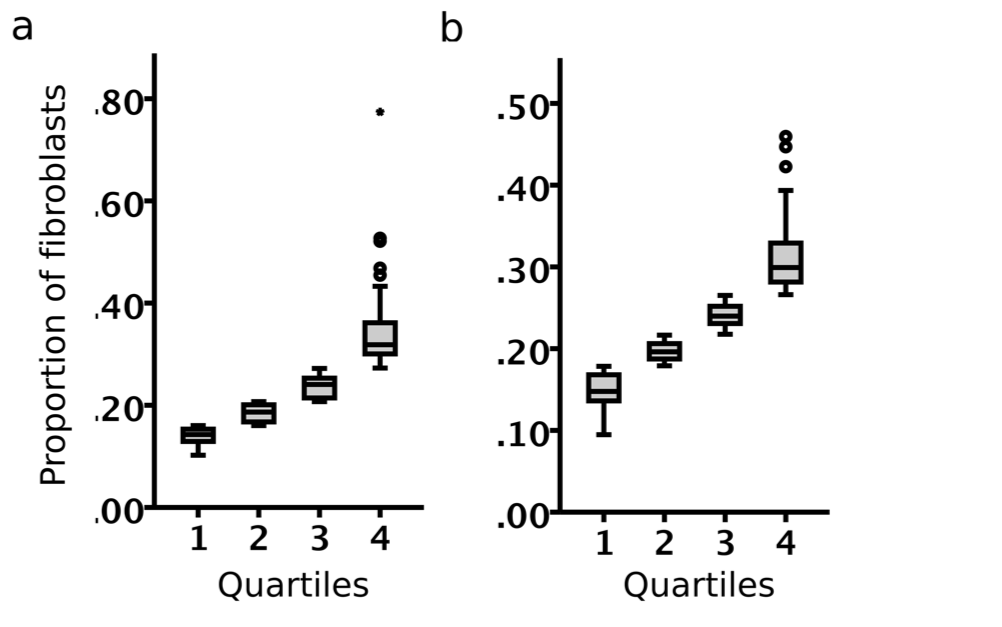


Figure S6. Proportion of stromal fibroblasts in (a) Cohort I (n=115) and (b) Cohort II (n=248). Boxplot indicates minimum, first quartile, median, third quartile, and maximum. Individual data points (circle and asterisk) are indicating outliers.


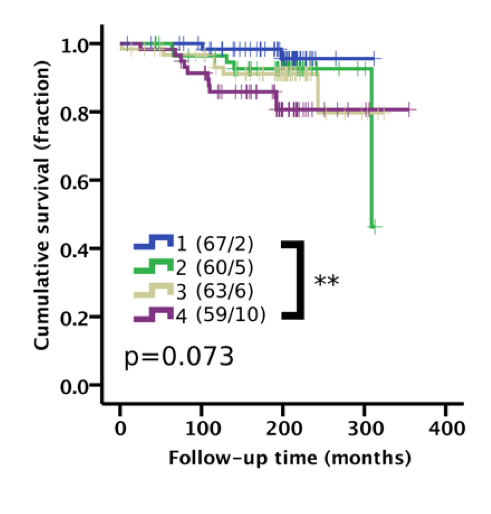


Figure S7. High proportion of fibroblasts in benign (adjacent to tumour) TMA cores predicts poor cancer-specific outcome in Cohort II (n=249). Kaplan-Meier analysis using quartiles (1–4) of the proportion of stromal fibroblasts. Number of patients and events are presented in brackets. Log-rank test, **p=0.009.


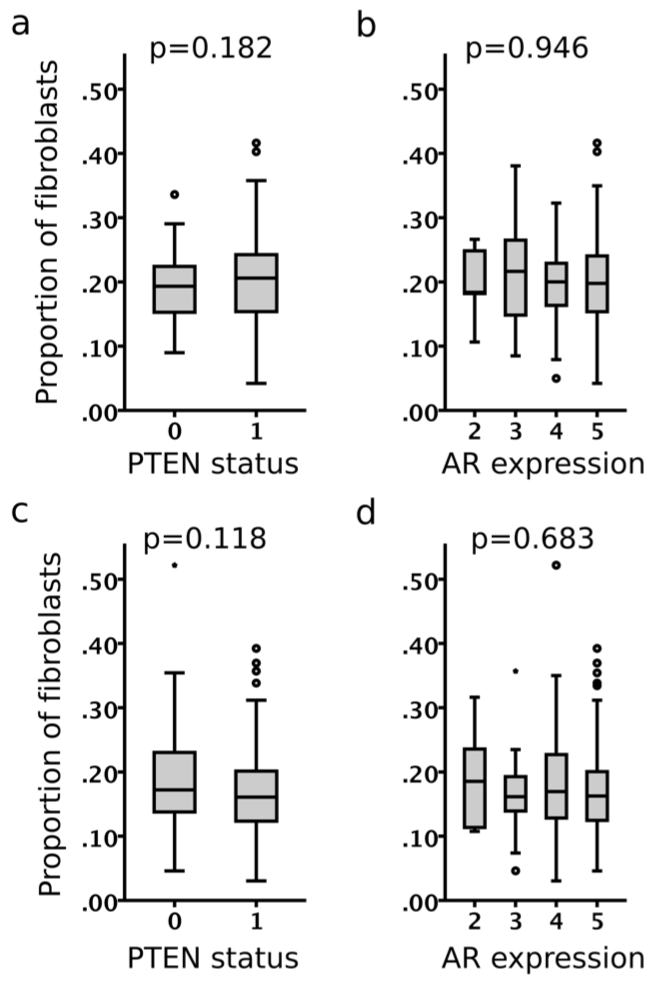


Figure S8. Association of the proportion of fibroblast with PTEN protein expression status (0=complete loss, 1=no loss) in (a) cancer (n=240 patients) and (c) benign TMA cores (n=241). Mann-Whitney U test. Association of the proportion of stromal fibroblast with maximum AR protein expression in (b) cancer (n=242) and (d) benign cores (n=243). Kruskal-Wallis H test. Boxplot indicates minimum, first quartile, median, third quartile, and maximum. Individual data points (circle and asterisk) are indicating outliers.


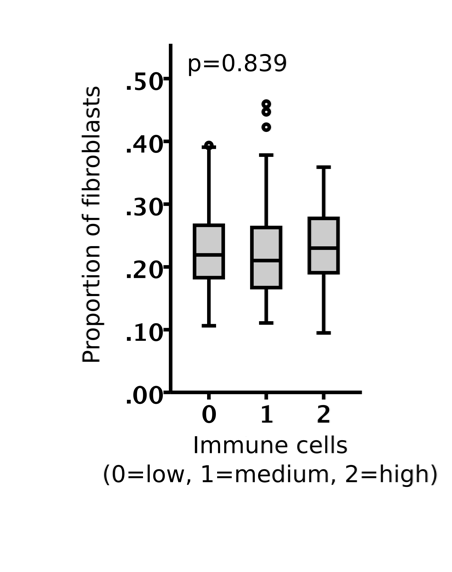


Figure S9. Distribution of fibroblasts (VIM-pos/aSMA-neg cells) in tumours with different amount of VIM-positive immune cells (visual assessment). Kruskal-Wallis H test. Boxplot indicates minimum, first quartile, median, third quartile, and maximum. Individual data points (circle) are indicating outliers. Cohort II (n=331).

Table S1. Antibodies used in the study. HIER, heat-induced epitope retrieval; IHC, immunohistochemistry; mIHC, multiplexed immunohistochemistry; ms, mouse; N.A., not applicable; rbt, rabbit; TSA, tyramide signal amplification; o/n, overnight.

Table S2. Demographics of patient cohorts including only patients with high-quality image data available. AR, androgen receptor; IQR, interquartile range; PTEN, phosphatase and tensin homolog.

Table S3. Hazard ratios (HR) in univariate and multivariate Cox Proportional Hazard regression analysis for prostate cancer-specific mortality (PCSM) in non-CRPC patients in Cohort I. HR is reported as per 10% change in the proportion of fibroblasts. Dx, diagnosis, Tx, treatment. Bolded p-values remain significant after Benjamini-Hochberg procedure. Wald test.
